# A Novel Method for the Localization and Management of Traumatic Cyclodialysis Cleft

**DOI:** 10.1155/2014/761851

**Published:** 2014-03-11

**Authors:** Mingling Wang, Shufang Hu, Zhenquan Zhao, Tianlin Xiao

**Affiliations:** ^1^Eye Hospital at Wenzhou, Wenzhou Medical College, 270 West Xueyuan Road, Wenzhou, Zhejiang 325027, China; ^2^Eye Hospital at Hangzhou, Wenzhou Medical College, 618 East Fengqi Road, Hangzhou, Zhejiang 310020, China

## Abstract

*Purpose.* To propose a novel surgical method for the localization and management of traumatic cyclodialysis clefts. *Methods.* Five patients with traumatic cyclodialysis clefts who underwent the innovative surgery were retrospectively reviewed. The new method was introduced to repair a cyclodialysis cleft with two running sutures from the middle to each end of the cleft under the guidance of a probe. Preoperative and postoperative visual acuity (VA), intraocular pressure (IOP), slit lamp and gonioscopic results, ultrasound biomicroscopy (UBM), and optical coherence tomography (OCT) findings were recorded. *Results.* Cyclodialysis clefts were completely closed postoperatively in four patients (four eyes); this was confirmed by progressively improved VA, restoration into the normal range of the IOP, disappearance of suprachoroidal fluid, and reduced macular edema. Only one patient with multiple clefts had an incomplete reattachment. *Conclusions.* This clinical study offers a novel and efficient method to localize and repair the cyclodialysis clefts.

## 1. Introduction

A cyclodialysis cleft is the result of the separation of the longitudinal ciliary muscle fibers from scleral spur and causes an abnormal drainage pathway for the aqueous humor [1, 2]. Blunt ocular trauma has been proposed to be the most common cause, followed by iatrogenic complications [3–5]. If complications, such as cataracts or macular edema, occur, exact localization and appropriate treatment are required. Multiple methods have been reported to identify and close cyclodialysis clefts [1, 6–10]. However, unsuccessful cases of cleft repair continue to be observed in clinical practice [11, 12].

To better manage the cyclodialysis cleft, we developed a novel method involving a probe to detect the exact extent of the cleft, as well as the use of two running sutures to fix the cyclodialysis cleft.

## 2. Methods

We retrospectively examined the clinical documentation of five consecutive patients with traumatic cyclodialysis clefts who were treated with the new surgical procedure in the Eye Hospital of Wenzhou Medical College from January 2010 to October 2012. This study was reviewed and approved by the Institutional Ethics Committee. Informed consent was obtained from all patients. The clinical records, including general information and examination results, were collected. The minimum follow-up period for the patients was six months postoperatively (Table 1). Reexamination of the patients consisted of a routine ophthalmologic examination in conjunction with fundus photography, gonioscopy, B-scan, OCT, and UBM.

### 2.1. Surgical Procedure

**Figure 1:**
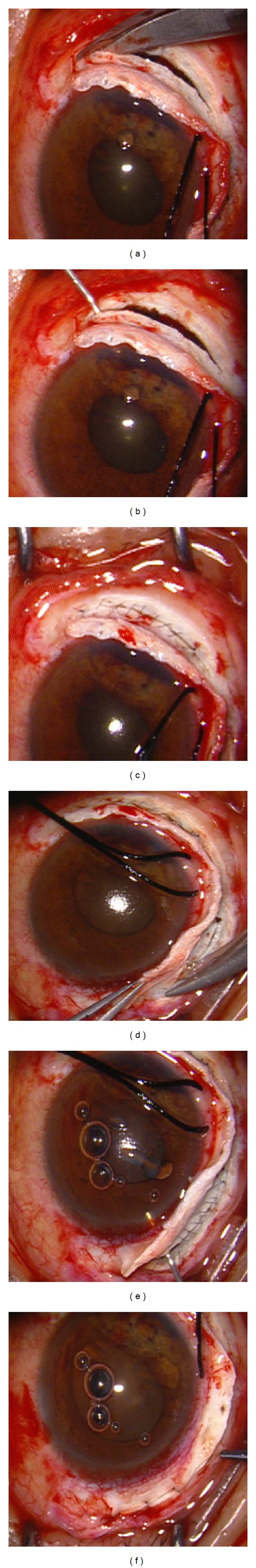
Surgical procedure to close the cyclodialysis cleft (case 3). (a) Beginning with a half deep scleral incision with scissors, we moved from the middle to the inferior end of the cleft behind the limbus, 1.5 mm, following the partial-thickness scleral flap. (b) A blunt probe was inserted into the anterior chamber, which was filled with a viscoelastic agent, and then the end of the cleft was gently detected. (c) Beginning with the first starting stitch from the middle of the cleft, we then extended to the end with a running 10-0 nylon suture. (d) Dissecting the other half of the deep scleral flap over the other half cleft. (e) Again detecting with the probe, we closed the cleft as described previously. (f) Closing the scleral flap and conjunctiva with interrupted sutures, respectively.

**Figure 2:**
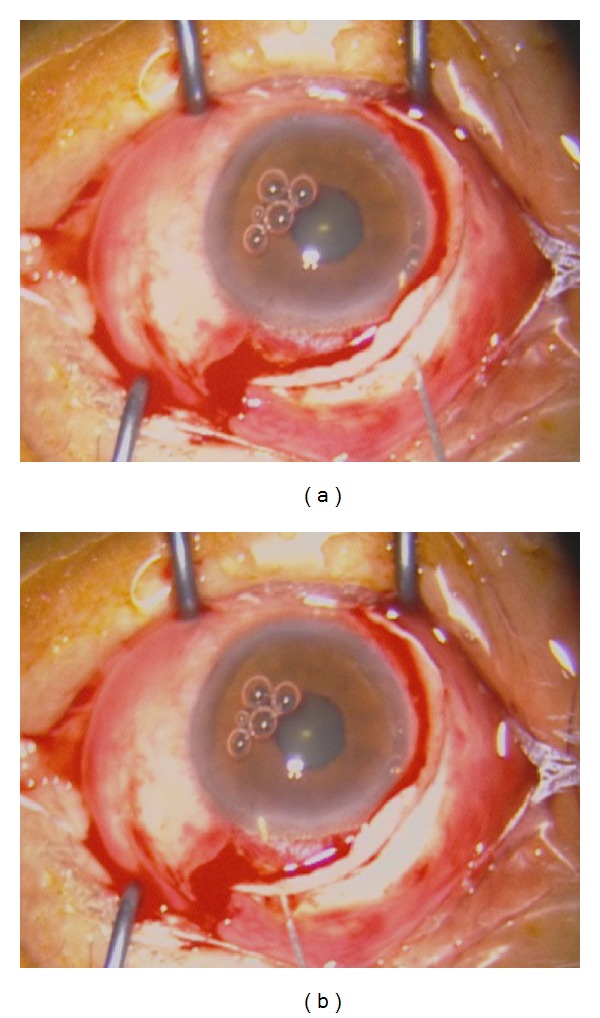
Probe detection of a cleft (case 5). A blunt needle with a syringe was gently inserted into the anterior chamber through a deep scleral incision from the middle (a) to the end of the cleft (b), stopping whenever it was unable to move. This stop site was the anatomical site of the ciliary muscle's attachment to the scleral spur, as well as the exact end of the cleft.

**Figure 3:**
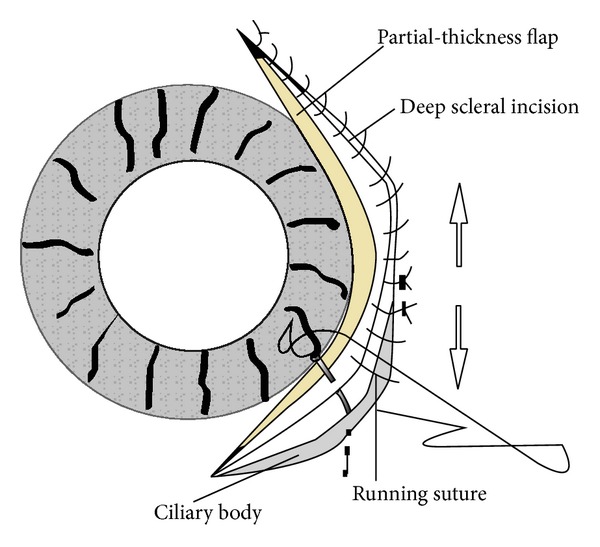
Deep scleral incision and running suture. A half of deep scleral incision is first made from the middle toward the end of the cleft and then closed with a running suture also starting from the middle to the end of the cleft. The same procedure is followed for the other half of the cleft (arrows show the direction of the suturing).

A miotic agent (0.5% pilocarpine) was applied before the surgery. A fornix-based conjunctival flap was created according to the location of a cyclodialysis cleft and then followed by a limbus-based, partial-thickness scleral flap at 3 mm from the limbus. A deep scleral incision, approximately half the length of the cleft, was first made from the middle toward one end of the cleft, 1.5 mm posterior and parallel to the limbus (Figure 1(a)). With the injection of a small amount of viscoelastic agent into the anterior chamber through the deep scleral incision, a probe (blunt needle) was inserted into the anterior chamber to explore the extent of the cleft from the middle (Figure 2(a)) gently toward the end of the cleft (Figures 1(b) and 2(b)), stopping whenever it was unable to go any further. This stop site was the exact end of the cleft. The deep scleral incision was then closed with a running 10-0 nylon suture, starting from the middle and extending to the end (Figures 1(c) and 3). This needle with a suture passed through the anterior scleral lip, the detached ciliary body, and finally the posterior scleral lip (Figure 3). The same procedure was performed for the other half of the cleft (Figures 1(d), 1(e), and 3). Finally, the partial-thickness scleral flap and conjunctiva were closed with interrupted sutures (Figure 1(f)).

## 3. Results

**Figure 4:**
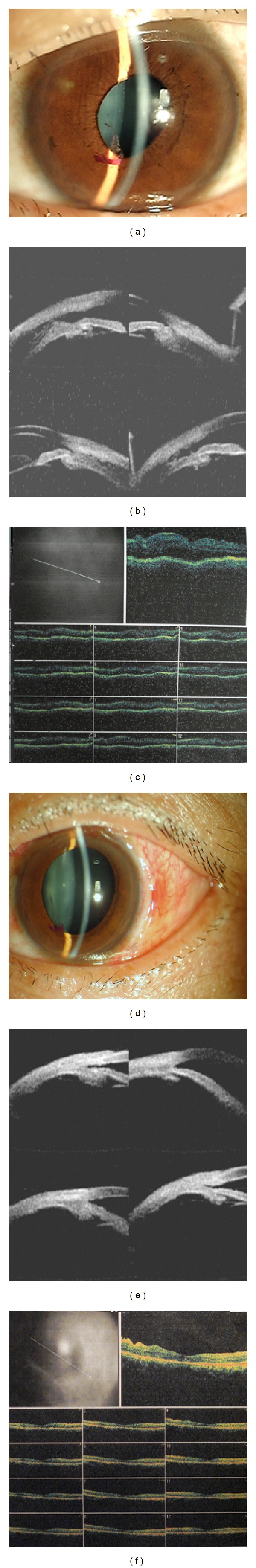
Anterior segment photography, UBM, and OCT in case 1. The top row represents the images before surgery. (a) A slightly shallow anterior chamber with vitreous prolapse. (b) A cleft and detached ciliochoroidal with suprachoroidal space effusion. (c) Retinal folds and macular edema. The bottom row is the images after surgery. (d) Recovery with a deeper anterior chamber and pharmaceutically dilated pupil. (e) Complete closure of the cyclodialysis cleft and the resolution of the ciliochoroidal detachment. (f) Presence of fovea centralis and reduced retinal folds.

The preoperative and postoperative IOPs and VAs of the patients were recorded in Table 2. The final IOP stayed within a normal range in four patients, although the fourth patient experienced slight ocular hypotension. At the postoperative reexamination, the VA improved in all patients. The anterior chamber became deep and quiet, with a decrease in macular edema and retinal folds (Figure 4). The fluid in the suprachoroidal space disappeared, and the ciliary body was reattached (Figure 4) in the first three and the fifth patients. A shallow suprachoroidal space and macular edema were still visible in the fourth patient, even though a closed cleft was observed upon UBM.

## 4. Discussion

Many techniques have been proposed to detect clefts preoperatively or intraoperatively. Gonioscopy, UBM, and AS-OCT have been recognized as the most reliable tools for the preoperative inspection of cleft conformations, particularly for the first two techniques. Those noninvasive methods are able to determine the clock-hour locations of a cleft and thus guide the cleft repair [13–15]. The limitations of gonioscopy or UBM in localizing a cleft emerge not only based on the influence of the examiner's judgments but also by a patient's situation, for example, head position, opacity of media, and depth of the anterior chamber. To address any possible underestimation from those influencing factors, an incision is recommended to be made 0.5–2 mm over the ends of the cleft [16]. For the intraoperative localization of a cleft, gonioscopy is used almost exclusively. As described above, intraoperative gonioscopic observation may also be influenced by certain circumstances. For instance, the location of the cleft in patient 3 was not clearly visualized intraoperatively by gonioscopy due to hyphema in the inferior angle.

Using a probe to detect the extent of a cleft is a novel technique that could provide the direct and exact localization of a cleft intraoperatively. Through this method, a probe can be easily inserted into the anterior chamber, freely moved toward the end of the cleft and stopped wherever the ciliary muscles are attached. This stop site is the exact end of the cleft. Therefore, we were able to mark both ends more accurately and suture them more correctly, avoiding insufficient or extra incisions or sutures. For example, the preoperative UBM finding in patient 2 showed a cleft between 12:30 and 4:00, but it was found to be exactly between 1:00 and 6:00 during intraoperative probe detection. Moreover, a probe could be used whenever a corneal edema, hyphema, or shallow anterior chamber was found (Figure 2). Because the probe can be moved in the anterior chamber through a cleft, the relaceration of the ciliary muscle or intraocular infection may be a consideration. However, these complications did not occur in our five patients, who were confirmed to have no bleeding or inflammatory cells in the anterior chamber during or after the operation. A few seconds of gentle movement in the anterior chamber by a probe regularly would not induce intraocular trauma or infection. The fact that a slightly larger cleft was detected by the probe than by UBM in this case series may not be due to injuries from probing. Instead, it is likely that the small linear ends of a cleft might not easily be detected by UBM or gonioscopy but could be accurately explored by probing. It seems that probe detection is more accurate and reliable. However, it should be noted that intraoperative probe detection must rely on or occur in conjunction with the results of preoperative gonioscopy and UBM. Furthermore, multiple clefts or incomplete cyclodialysis is not suitable for probe detection.

The management of a cyclodialysis cleft is aimed at reaffixing the detached ciliary body to the internal scleral surface. Among various surgical interventions, direct cyclopexy has been widely used by many surgeons all over the world [17–20]. The conventional direct cyclopexy technique requires a lot of time to repeat the incision-suture process, and the visualization of whether the detached ciliary body had been sutured was restricted due to the neighboring interrupted knots' interference with the visualization. The technique we described in this paper involved the reattachment of the ciliary body successfully with two running sutures to close each half of the cleft. Its excellent advantages included the time saved due to the use of only two running sutures, which also benefitted the visualization of whether the suture was passed through the detached ciliary body. An unsutured cleft may be another reason for failed surgical cases. Running suture fixation, combined with probing detection, can be accomplished concurrently from the middle to the end of a cleft; thereby, it is able to extend wherever the incision or suture is needed (Figure 3).

With this novel method, four patients achieved a successful anatomical and functional recovery. These patients presented with improved VA, increased IOP, and decreased macular edema (Table 2, Figure 4). The slight hypotony and shallow suprachoroidal fluid remained in the fourth patient with multiple clefts; this case may indicate that a potential poorly closed cleft existed and further follow-up or management may be needed.

All of the five patients who underwent our new surgery presented with transient hypertension on the first postoperative days, with the duration varying from day 1 to day 7 (mean 3.5 days) and with IOP spike varying from 26 to 60 mmHg. All of the ocular hypertension was medically controlled. No wound rupture or glaucomatous cupping of the optic nerve head was noted.

Overall, the novel method described in this paper exhibited two remarkable advantages: the direct and exact intraoperative localization of a cleft, regardless of the cloudiness of the anterior chamber or cornea, and the simplicity of using only two running sutures to close each half of the cleft. Our clinical practice has shown that this technique is simple, safe, and efficient; however, further repetitions of this technique and its use on chronic cases should be evaluated to assess its long-term efficiency and safety.

## Figures and Tables

**Table 1:** General information on the five patients with cyclodialysis clefts.

No.	Sex/ages	OD/OS	Causes	Extent of cleft		Follow-up
				UBM or gonioscopy	Probe	
1	M/37 yrs	OS	Grindstone	12:00–4:00	12:00–5:00	6.5 mo
2	M/23 yrs	OS	High pressure gas	12:30–4:00	1:00–6:00	6 mo
3	M/37 yrs	OS	High pressure water gun	6:00–10:30	5:30–12:00	14 mo
4	F/50 yrs	OD	Stone	12:00–1:00	11:00–12:30	8 mo
				1:30–3:00	1:00–3:00	
5	M/56 yrs	OS	Apple	7:00–12:00	7:00–1:00	9 mo

M: male; F: female; yrs: years; mo: month; d: day.

**Table 2:** IOP and VA of the five patients with cyclodialysis clefts.

Case no.	IOP (mmHg)		VA	
	Preoperative	Postoperative	Preoperative	Postoperative
1	6.7 ± 0.5	16.4 ± 3.6	FC/30 cm	0.3
2	6.9 ± 0.2	14.0 ± 2.1	0.06	0.2
3	5.0 ± 0.6	13.0 ± 3.5	HM/50 cm	0.1
4	6.1 ± 0.3	8.4 ± 0.8	0.12	0.4
5	7.1 ± 0.7	15.6 ± 1.8	FC	0.2

IOP: intraocular pressure; VA: visual acuity; FC: finger count; HM: hand motion.
